# Leaves from My Experience

**Published:** 1889-02

**Authors:** J. A. Robinson


ARTICLE V.

### LEAVES FROM MY EXPERIENCE.

BY J. A. ROBINSON, D. D. S.

Some five months ago, I filled seven cavities in the labial surface in front incisors quite under the free margin of the gums for a lady who appeared well, and about thirty years old. She came because I used an obtunder for preparing the cavities. I got along pretty well with the operation and she seemed pleased. She paid the bill in part, which was $30, and left a balance for two months. At the end of the two months she paid half the balance and complained that some of the fillings were turning dark, and thought the gold was not pure. As the teeth were filled within two days. I told her the gold was the same in all the teeth, and enquired if she had been taking any medicine, and she told me "she *had not*." I told her that forty years ago, when we finished all fillings by burnishing, the fillings sometimes turned brown by the rubbing of the iron from the instrument, and I knew of nothing that would act in that way upon pure gold but iron and sulphur. She had taken nothing of the kind. As there was quite a number of cavities in the lower teeth to be filled that presented the same appearance of those that had been filled. I insisted that I thought she must have been taking some medicine that had turned the fillings, and perhaps was the original cause of the caries in her teeth. I afterward learned from a talk with her husband that she had been taking Fellow's Hypophosphite for more than three years.

The medicine was taken for nervousness, and she had received great benefit from the medicine, but perhaps it had injured her teeth. The mineral substance of hypophosphite is manganese and iron; both substances are strong in coloring qualities, and manganese has been used in the coloring of the enamel of artificial teeth a dark brown, like that on the gold fillings. I have known of two instances where the enamel was destroyed in two months by the use of muriate of iron. It will turn crown work quite dark in a few days made of *coin gold.* Packing the gums at night with prepared chalk (carbonate of lime) to be brushed off in the morning, will keep such fillings always bright, and will not injure the mouth, and the patients can do it themselves. As crown and bridge-work are fast coming to the front in operative dentistry, it may not be amiss to describe a short and easy way to repair them in case of accident. To remove crowns, I cut down the backing, if the tooth is broken on either side till it is very narrow, and with a small drill, drill a half dozen holes through the top or end of the crown inside of the band, then with a small fissure burr cut each hole into the next hole inside the band until it is entirely free all round. If the post is a screw it can be unscrewed and the band easily removed, and a new top or end put on and the tooth replaced in a very short time without pain. In bicuspids, with bifercated roots, I drill a small hole into each root, then make a staple or loop from a small hair pin, and cement into each hole, leaving it long enough to hold the oxyphosphate. In this way crowns can be made strong where the roots are decayed below the gums. Justi's cement is very strong but slow setting, so I mix about equal parts of Sampson cement with Justi's as it saves time and seems equally as strong. The same old discussions are going on at all the meetings about dental patents, and professional etiquette, and the donations of all the new improvements to the profession. It will be a happy time when physicians and dentists learn to do business on business principles. Love of approbation and encomiums are cheap, and it is gratifying to ones' friends to hear them spoken well of when they have passed away; but the time has gone by when people enjoy the abuse of Zantippe, the wife of Socrates, and call her an intolerable scold for talking back to Socrates, who never gave her a shilling to mend her shoes or provided a dinner for his family. Martyrs of to-day are the heroes of to-morrow. Fifty years ago the dentists in New England were mostly M. D.'s, but as manipulative dentistry is mechanism of the finer sort, combined with taste and gentleness, so almost all the improvements in our instruments and appliances have been perfected by the skill of those who obtained their education as best they could, and had adaptation and consecration and love for their calling. Professor Taft says the discussion is unprofitable, which is undoubtedly true; but he once said in public that he had never known a first-class operator in dentistry who was not a dentist before he became an M. D. Medicine will be as effectual, and appliances as useful if *good for any thing*, whether they are marked patent or donated to the profession. A rounded man, and a rounded life are magnificent things to behold, but true manhood is never attained by titles or diplomas or casts or sects.—*Cincinnati Medical Journal.*

